# Gut Microbial Postbiotics as Potential Therapeutics for Lymphoma: Proteomics Insights of the Synergistic Effects of Nisin and Urolithin B Against Human Lymphoma Cells

**DOI:** 10.3390/ijms26146829

**Published:** 2025-07-16

**Authors:** Ahmad K. Al-Khazaleh, Muhammad A. Alsherbiny, Gerald Münch, Dennis Chang, Deep Jyoti Bhuyan

**Affiliations:** 1 NICM Health Research Institute, Western Sydney University, Penrith, NSW 2751, Australia; 19316068@student.westernsydney.edu.au (A.K.A.-K.); d.chang@westernsydney.edu.au (D.C.); 2 Pharmacognosy Department, Faculty of Pharmacy, Cairo University, Cairo 11562, Egypt; muhammad.alsherbiny@pharma.cu.edu.eg; 3 Freedman Foundation Metabolomics Facility, Innovation Centre, Victor Chang Cardiac Research Institute, Sydney, NSW 2010, Australia; 4 Pharmacology Unit, School of Medicine, Western Sydney University, Campbelltown, NSW 2560, Australia; g.muench@westernsydney.edu.au; 5 School of Science, Western Sydney University, Penrith, NSW 2751, Australia

**Keywords:** lymphoma, postbiotics, Nisin, Urolithin B, synergy, proteomics, apoptosis

## Abstract

Lymphoma continues to pose a significant global health burden, highlighting the urgent need for novel therapeutic strategies. Recent advances in microbiome research have identified gut-microbiota-derived metabolites, or postbiotics, as promising candidates in cancer therapy. This study investigates the antiproliferative and mechanistic effects of two postbiotics, Nisin (N) and Urolithin B (UB), individually and in combination, against the human lymphoma cell line HKB-11. Moreover, this study evaluated cytotoxic efficacy and underlying molecular pathways using a comprehensive experimental approach, including the Alamar Blue assay, combination index (CI) analysis, flow cytometry, reactive oxygen species (ROS) quantification, and bottom-up proteomics. N and UB displayed notable antiproliferative effects, with IC_50_ values of 1467 µM and 87.56 µM, respectively. Importantly, their combination at a 4:6 ratio demonstrated strong synergy (CI = 0.09 at IC_95_), significantly enhancing apoptosis (*p* ≤ 0.0001) and modulating oxidative stress. Proteomic profiling revealed significant regulation of key proteins related to lipid metabolism, mitochondrial function, cell cycle control, and apoptosis, including upregulation of COX6C (Log_2_FC = 2.07) and downregulation of CDK4 (Log_2_FC = −1.26). These findings provide mechanistic insights and underscore the translational potential of postbiotics in lymphoma treatment. Further preclinical and clinical investigations are warranted to explore their role in therapeutic regimens.

## 1. Introduction

Lymphoma, a heterogeneous group of malignancies originating from lymphocytes, remains a significant global health burden despite advancements in cancer research and treatment modalities. According to recent global cancer statistics, lymphoma ranks among the ten most common cancers worldwide, with an increasing incidence and mortality rate [1]. Non-Hodgkin’s lymphoma (NHL) and Hodgkin’s lymphoma (HL) are the two primary subtypes, characterised by distinct clinical and molecular features. Moreover, while advancements in immunotherapy and targeted treatments have improved outcomes for many patients, the prognosis for aggressive or refractory lymphoma remains suboptimal, necessitating novel therapeutic approaches [2,3].

HKB-11, a hybrid cell line that exhibits key characteristics of non-Hodgkin B-cell lymphoma, serves as a relevant model for investigating therapeutic interventions. Also, HKB-11 shows a doubling time of approximately forty-one hours in serum medium, with similar growth rates in chemically defined formulations, confirming its robust proliferation across various culture systems [4]. For instance, Mei and colleagues demonstrated that HKB-11 supports eight to thirty times higher factor VIII secretion than HEK293 or BHK21, highlighting its efficient endoplasmic reticulum trafficking and secretory capacity [5]. Moreover, HKB-11 can grow to high densities in serum-free suspension without aggregation, ensuring uniform drug exposure during viability, apoptosis, and proteomics assays [4,6]. Additionally, these characteristics establish HKB-11 as a scalable human Burkitt lymphoma model that encapsulates MYC dependence, EBV latency, rapid growth, and uncomplicated assay logistics, justifying its selection for antiproliferative and synergy studies while exploring novel therapies.

Emerging evidence suggests a critical role for the gut microbiota in modulating cancer pathogenesis, treatment responses, and patient outcomes. The gut microbiota comprises a complex ecosystem of bacteria, fungi, archaea, and viruses that interact with the host to maintain homeostasis and influence various physiological processes, including immune regulation, metabolism, and inflammatory responses [7]. Recent studies highlighted the intricate relationship between gut microbial dysbiosis and lymphoma, suggesting that specific microbial metabolites may modulate tumour growth, immune evasion, and responses to therapy [8,9,10]. Gut microbial metabolites, often called postbiotics, represent bioactive molecules derived from microbial metabolism that may exhibit diverse biological functions, including anticancer and immunomodulatory activities [11,12,13]. Nonetheless, studies investigating the interactions of postbiotics with cancer and other diseases are still in their early stages and warrant further exploration.

Among the most studied postbiotics are inosine, Nisin (N), Urolithin A (UA), Urolithin B (UB), and short-chain fatty acids (SCFAs), which have shown potential therapeutic benefits in various cancer models [14,15,16,17]. Inosine, a purine metabolite, has demonstrated immunostimulatory effects by enhancing T-cell activation and modulating the tumour microenvironment [14]. N, a bacteriocin produced by certain strains of *Lactococcus lactis*, has exhibited antiproliferative and pro-apoptotic effects in in vitro and in vivo cancer studies, including lymphoma models [15,16]. Urolithins, metabolites derived from dietary ellagitannins through gut microbial metabolism, have shown anti-inflammatory and anticancer properties, particularly by inducing apoptosis and modulating cell signalling pathways in haematological malignancies [17,18,19,20]. SCFAs, primarily acetate, propionate, and butyrate, are by-products of the fermentation of dietary fibres by gut bacteria. They have been extensively studied for their role in immune modulation, epigenetic regulation, and tumour suppression [21,22,23,24,25]. The mechanisms through which these metabolites exert their anticancer effects can be multifaceted, involving the modulation of immune responses, alteration of cell signalling pathways, and induction of programmed cell death [22,26]. For instance, butyrate has been shown to inhibit histone deacetylases (HDACs), leading to epigenetic reprogramming and apoptosis in lymphoma cells [27,28,29]. Similarly, UA has been reported to enhance the efficacy of chemotherapeutic agents by inhibiting key pathways associated with cell proliferation and survival in colorectal cancer cells [30,31]. Despite these promising findings, the molecular mechanisms underlying the antiproliferative effects of these metabolites against lymphoma remain inadequately explored. Against this backdrop, proteomics offers a robust and comprehensive approach to characterise cellular responses, enabling deeper insights into the pathways and molecular networks modulated by these metabolites.

In this context, the current study investigated the antiproliferative activities of postbiotics, including inosine, N, UA, UB, and SCFA salts, in cellular models of lymphoma. The study also evaluated the potential synergistic effects of these metabolites when combined. By employing advanced proteomic and cell-based approaches, we elucidated the molecular mechanisms underlying their anticancer activities and identified potential biomarkers for therapeutic targeting.

## 2. Results and Discussion

### 2.1. Antiproliferative Activity of the Seven Postbiotics Against the HKB-11 (BL) Human Cell Line

The antiproliferative activities of seven postbiotics, including magnesium acetate, sodium propionate, sodium butyrate, inosine, N, UA, and UB, were evaluated against HKB-11 lymphoma cells over 72 h using the Alamar Blue assay (Table 1).

Among these, N (IC_50_ = 1467 µM) and UB (IC_50_ = 87.56 µM) demonstrated the most potent inhibitory effects (*p* ≤ 0.05). The comparative analysis of antiproliferative effects against HKB-11 lymphoma cells revealed statistically significant differences among the tested metabolites. The IC_50_ of N alone was relatively high (~1467 µM), raising concerns about clinical feasibility. However, when combined at a 4:6 ratio, strong synergy was achieved at substantially lower concentrations, specifically N at 3200 µM and UB at 300 µM (CI = 0.09 at IC_95_). N is a 34-amino-acid lantibiotic peptide produced by *Lactococcus lactis* during fermentation, with negligible endogenous production in humans [32]. It has been safely used as a food preservative for over 50 years, with GRAS (Generally Recognised as Safe) status by the U.S. FDA and approval by the EFSA at levels of 1–12.5 mg/kg in food products [33]. However, its systemic therapeutic application is limited by its peptide nature, ~3.5 kDa size, and susceptibility to proteolytic degradation, which restricts oral bioavailability and systemic exposure [34]. These findings highlight the established safety profile of N and underscore the need for alternative delivery strategies or local administration to achieve pharmacologically relevant levels in vivo. Our study provides proof-of-concept evidence that combining N with UB can reduce the required concentrations and achieve potent antiproliferative activity in lymphoma models, warranting further pharmacokinetic and animal studies to assess feasibility. Further pharmacokinetic and bioavailability studies are warranted to assess whether these doses are attainable in vivo.

For instance, at 8000 µM, N showed complete inhibition of cell growth (100.26 ± 0.09%), which was significantly greater (*p* ≤ 0.05) than sodium butyrate (57.83 ± 9.95%), sodium propionate (38.07 ± 10.39%), magnesium acetate (9.97 ± 3.12%), and inosine (30.91 ± 6.27%).

Moreover, similar trends were observed at 4000 µM, where N maintained total inhibition (100.21 ± 0.18%), statistically exceeding sodium butyrate, sodium propionate, magnesium acetate, and inosine. Furthermore, at 2000 µM, N (65.22 ± 1.35%) showed similar activity to that of sodium butyrate (45.36 ± 9.08%; *p* ≥ 0.05), and greater activity compared to sodium propionate, magnesium acetate, and inosine (*p* ≤ 0.05). This trend continued at 1000 and 500 µM of N. All postbiotics exhibited dose-dependent activity, exhibiting greater efficacy at higher doses.

As a bacteriocin, N has a well-established role in antimicrobial applications, but its anticancer potential has recently been gaining recognition. Studies have demonstrated that N induces apoptosis in cancer cells through mitochondrial membrane disruption, the generation of ROS, and the modulation of pathways such as caspase activation and autophagy [25,35,36,37]. For example, Shen et al. (2018) reported that N significantly inhibited the proliferation of head and neck squamous cell carcinoma cells by increasing intracellular ROS and downregulating the Akt/mTOR signalling pathway [35]. Furthermore, N’s ability to target cancer stem cells and enhance the efficacy of conventional chemotherapeutic agents such as doxorubicin makes it a desirable candidate for further research [36,37]. Recent research by our group has provided additional mechanistic insights into the antiproliferative action of N in breast adenocarcinoma [25]. Specifically, N induced significant apoptotic cell death in breast adenocarcinoma cell lines (MCF7 and MDA-MB-231) by modulating ROS production and potentially triggering cell cycle arrest [25]. Furthermore, proteomic analyses revealed alterations in proteins associated with cell cycle regulation, apoptosis, and cellular stress responses, indicating a multifaceted mode of action [25]. N’s impact on these cellular processes suggested that its anticancer effects extend beyond simple cytotoxicity, as it also interferes with the molecular machinery required for cancer cell survival and proliferation [25]. These findings underline N’s potential as a therapeutic agent, particularly in combination with other postbiotics or chemotherapeutic agents, to exploit synergistic effects and maximise efficacy.

UB also demonstrated significantly greater antiproliferative activity across all nine tested doses compared to UA (*p* ≤ 0.05; Table 1). For instance, at 500 µM, UB inhibited cell growth by 81.81 ± 9.51%, significantly greater than UA (61.51 ± 13.44%; *p* ≤ 0.05). This trend continued even at the lowest tested concentration of 1.953 µM. The IC_50_ values supported these findings, with UB showing the greatest potency with an IC_50_ of 87.56 µM, followed by UA at 384.41 µM; both were substantially more active than N (1467 µM), sodium butyrate (2022 µM), and sodium propionate (14,597.14 µM). UB has been previously shown to induce apoptosis, impair cell proliferation, and modulate key signalling pathways in cancer cells. For instance, studies have demonstrated that UB can inhibit the NF-κB pathway, reducing inflammation and enhancing apoptosis in breast and colorectal cancer models [38]. Additionally, its ability to induce oxidative stress and mitochondrial dysfunction in cancer cells further underscores its potential [39]. UA, though less potent than UB, has been associated with beneficial effects on mitochondrial biogenesis and autophagy, which may contribute to its anticancer properties [40].

The IC_50_ values of magnesium acetate and inosine could not be calculated due to insufficient antiproliferative activity at the tested doses (≤50%). Magnesium acetate consistently produced the weakest activity, with no apparent dose-dependent response, and was significantly less effective than the other treatments across all tested concentrations. Also, among the tested SCFAs, sodium butyrate demonstrated significantly greater activity (*p* ≤ 0.05) and a clear dose–response relationship in comparison to sodium propionate and magnesium acetate. Sodium butyrate was reported previously for its role as an HDAC inhibitor, inducing cell cycle arrest and apoptosis in various cancer types [41,42,43,44]. Overall, sodium propionate, magnesium acetate, and inosine exhibited limited efficacy in this study, suggesting a lower therapeutic priority for lymphoma models. The significant antiproliferative effects of N and UB against HKB-11 lymphoma cells highlight their potential as therapeutic agents. N’s strong efficacy and established safety profile make it an excellent candidate for immediate preclinical studies. Moreover, with its potent activity at low concentrations, UB provides a complementary avenue for therapeutic exploration. Therefore, the promising activities of N and UB make them suitable candidates for combination therapy.

### 2.2. The Synergy of N with UB Against HKB-11 Lymphoma Cells

Nine different ratios of N and UB, 1:9 *v*/*v* (800:450 μM), 2:8 *v*/*v* (1600:400 μM), 3:7 *v*/*v* (2400:350 μM), 4:6 *v*/*v* (3200:300 μM), 5:5 *v*/*v* (4000:250 μM), 6:4 *v*/*v* (4800:200 μM), 7:3 *v*/*v* (5600:150 μM), 8:2 *v*/*v* (6400:100 μM), and 9:1 *v*/*v* (7200:50 μM), were evaluated using Alamar Blue against HKB-11 lymphoma cells. Synergistic interactions (CI values ≤ 1; Table 2) were observed between N and UB between IC_75_ and IC_95_ at all combinations. At the 4:6 combination (N 3200 μM and UB 300 μM), the CI value was observed to be as low as 0.09 at IC_95_ (Table 2).

Following the synergy study on HKB-11 human lymphoma cells, the most potent synergistic combination, N and UB at the 4:6 ratio, was also further evaluated for its antiproliferative effects against Hs 313.T human lymphoma and HS-5 normal stromal cells (Table 3). The aim was to assess antiproliferative potency, selectivity, and therapeutic potential. The combination exhibited a dose-dependent inhibitory effect on both lymphoma cell lines. At the highest tested concentration of 3500 μM, the combination showed statistically similar cell growth inhibition values against both the HKB-11 and Hs 313.T cell lines (*p* ≥ 0.05; Table 3). Overall, Hs 313.T was more sensitive to the combination, with a lower IC_50_ of 335.4 μM, than HKB-11 cells (IC_50_ = 1304 μM). Specifically, at lower concentrations (1750, 875, and 437.5 μM), the combination exhibited significantly greater inhibition of Hs 313.T cells than HKB-11 cells (*p* ≤ 0.05; Table 3).

The impact of the combination on HS-5 normal stromal cells also followed a dose-dependent response (IC_50_ = 551.6 μM). At 3500 μM, cell viability was significantly reduced to 10.08 ± 4.01%, indicating cytotoxicity (Table 3). However, at lower concentrations, stromal cell viability improved markedly, reaching 92.32 ± 7.72% at 109.375 μM. At intermediate concentrations, such as 437.5 μM, stromal cell viability (66.37 ± 11.53%) remains high while the growth of Hs 313.T lymphoma cells is inhibited by 76.06 ± 4.43% (Table 3), highlighting a potential therapeutic window where cancer cells are effectively targeted while normal cells are spared [45,46]. Furthermore, the combination’s selectivity was further underscored by its lower IC_50_ against Hs 313.T, making it a promising candidate for subtype-specific therapies. The Hs 313.T line consistently showed higher sensitivity to both combinations, implying a biological distinction potentially rooted in genetic or metabolic vulnerabilities. Additionally, the significantly lower IC_50_ values for Hs 313.T underscored the possibility of subtype-selective efficacy. Such differential sensitivity is a known phenomenon in cancer pharmacology and reflects the heterogeneous nature of tumour biology and potential variations in cellular mechanisms or metabolic responses between the two cancer cell lines [47,48]. It carries an MYC rearrangement, mimicking the behaviour of high-grade B-cell lymphoma [5]. Moreover, HKB-11 cells are more likely to exhibit aggressive or proliferative traits, making them a better model for studying oncogenic signalling, resistance, and treatment-induced pathway modulation. HKB-11 cells are advantageous due to their stable growth and representation of certain Hodgkin-like features, including CD30 expression and responsiveness to apoptotic stimuli [49]. To complement this, we included Hs 313.T lymphoma cells (Table 3), which are derived from a diffuse large B-cell lymphoma (DLBCL) and exhibit phenotypic and genetic characteristics closer to aggressive human B-cell lymphomas, making them a valuable model for assessing therapeutic selectivity and efficacy [50]. However, we recognise that established and widely used B-cell lymphoma lines such as Raji (Burkitt lymphoma) and SU-DHL-4 (DLBCL) could provide additional validation and enable comparison with data from many other studies, as these lines are well-characterised and commonly employed in preclinical lymphoma research [51].

Overall, these results suggested a dose-dependent inhibition pattern in the two lymphoma cell lines tested. Both N and UB face challenges regarding bioavailability. N is degraded in the gastrointestinal tract and exhibits low systemic absorption [52], while UB shows moderate oral bioavailability but is extensively metabolised [53]. Thus, formulation strategies or delivery systems to enhance systemic exposure warrant exploration in future studies.

### 2.3. ROS Production in HKB-11 Lymphoma Cells After Treatment with Different Concentrations of N, UB, and N/UB (4:6)

**Figure 1:**
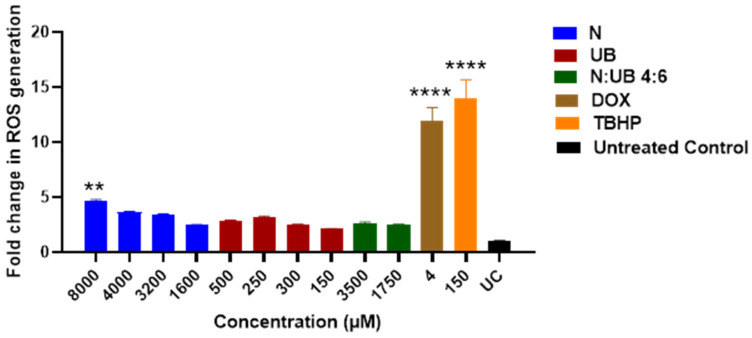
Modulation of reactive oxygen species (ROS) production in HKB-11 lymphoma cells. Cells were treated for 4 h with Nisin (N), Urolithin B (UB), or their 4:6 combination (N/UB) at the indicated concentrations. ROS levels were quantified using the H_2_DCFDA assay. Data are presented as the fold change in fluorescence relative to the untreated control (UC). Doxorubicin (Dox; 4 µM) and tert-butyl hydroperoxide (TBHP; 150 µM) were used as positive controls for ROS induction. Values represent the mean ± SD (*n* = 3). Statistical significance was determined by ANOVA with Dunnett’s post hoc test (** *p* ≤ 0.01; **** *p* ≤ 0.0001 compared to the untreated control). High-dose Nisin significantly increased ROS, while the N/UB combination resulted in a more balanced oxidative profile, similar to the untreated control.

Oxidative stress, characterised by an increase in ROS, is a hallmark of carcinogenesis, playing a pivotal role in cancer initiation, progression, and metastasis [54,55]. Inhibiting ROS production is a promising strategy for developing anticancer drugs [56]. This study investigated the effects of N, UB and their synergistic combination (4:6) on oxidative stress in HKB-11 lymphoma cells (Figure 1). The results are expressed as fold changes in ROS levels, providing insight into the potential of these compounds to modulate oxidative stress. N, UB, and their combination (N/UB) at the highest concentrations reduced ROS production in HKB-11 lymphoma cells compared to the established ROS inducer, tert-butyl hydroperoxide (TBHP), as shown in Figure 1. Doxorubicin (Dox) was included for comparative purposes.

Treatment with N alone, at concentrations of 8000 μM, resulted in a statistically significant increase in ROS levels compared to the untreated control (*p* ≤ 0.01), emphasising N’s strong oxidative potential. Furthermore, this dose-dependent rise of ROS supported the hypothesis that N possesses pro-oxidative activity, potentially inducing oxidative-stress-mediated apoptosis in lymphoma cells. Elevated ROS levels can disrupt mitochondrial function, induce DNA damage, and promote cancer cell death [54,57]. In contrast, UB administered alone (at 500 μM and 250 μM) induced only minimal changes in ROS levels, which were statistically comparable to those of the untreated control (*p* ≥ 0.05). This suggested that UB may have a neutral or possibly antioxidant role, consistent with findings that some compounds help modulate oxidative environments without directly elevating ROS [58].

Interestingly, the combination of N and UB (4:6; 3500 μm N/1750 μm UB) showed a modest increase in ROS levels; however, this increase was statistically similar to that of the untreated control (*p* ≥ 0.05). Also, the positive controls, Dox (4 μM) and TBHP (150 μM), induced high ROS production, validating the assay’s sensitivity and serving as benchmarks for oxidative stress induction. Dox is well-known for its redox cycling properties and mitochondrial disruption, which leads to cell death [59]. Additionally, these results have important therapeutic implications. The strong ROS induction by N at 8000 μM alone suggested its potential use as a pro-oxidative agent in cancer therapy, capitalising on the vulnerability of cancer cells to oxidative stress [55].

Moreover, when combined, these distinct but converging actions, N’s direct membrane disruption and ROS elevation and UB’s impairment of mitochondrial respiration and metabolic regulation, likely overwhelm the antioxidant defences of lymphoma cells, resulting in enhanced oxidative stress, mitochondrial dysfunction, and the activation of caspase-dependent apoptosis pathways. Such complementary targeting of mitochondrial integrity and metabolism represents a rational strategy for inducing cancer cell death while potentially reducing the required doses of each agent.

Meanwhile, the antioxidative effect of UB in combination therapy could help mitigate collateral damage to normal cells, offering a more selective and safer treatment strategy. In summary, our findings demonstrated that while N can significantly elevate ROS at higher doses and potentially induce apoptosis in cancer cells, its combination with UB provides a balanced oxidative profile. This approach may enable effective tumour targeting with reduced toxicity, thereby supporting the development of synergistic combination therapies for lymphoma.

### 2.4. Flow Cytometric Analyses of Apoptotic Profiles of HKB-11 Lymphoma Cells After Treatment with Different Concentrations of N, UB, and N/UB (4:6)

**Figure 2:**
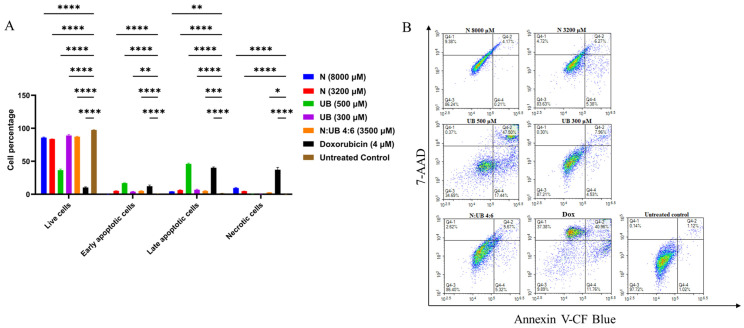
Synergistic induction of apoptosis in HKB-11 lymphoma cells by Nisin and Urolithin B. Cells were treated for 24 h with Nisin (N), Urolithin B (UB), their 4:6 combination (N/UB), or doxorubicin (Dox) as a positive control. Apoptosis was assessed by flow cytometry using Annexin V-CF Blue and 7-AAD staining. (**A**) Bar chart quantifying the percentage of cells in each population: live (Annexin V-/7-AAD-), early apoptotic (Annexin V+/7-AAD-), late apoptotic (Annexin V+/7-AAD+), and necrotic (Annexin V-/7-AAD+). Data are mean ± SD (*n* = 6). Statistical significance compared to the untreated control is denoted by * *p* < 0.05, ** *p* < 0.01, *** *p* < 0.001, and **** *p* < 0.0001. (**B**) Representative density plots for each treatment. The x-axis shows Annexin V-CF Blue fluorescence, and the y-axis shows 7-AAD fluorescence. The combination therapy significantly increased both early and late apoptosis compared to the individual treatments, while inducing less necrosis than doxorubicin.

Flow cytometry was used to assess the apoptotic responses in HKB-11 lymphoma cells following 24 h exposure to the most potent levels of N (8000 μM) and UB (500 μM) (chosen based on the findings of the Alamar Blue and ROS assays) and the most potent synergistic combination, 4:6 (3500 μM), and its individual components N (3200 μM) and UB (300 μM). As shown in Figure 2A, N at 8000 μM significantly increased cell death relative to untreated controls (*p* ≤ 0.01), with marked elevations in both apoptotic and necrotic populations. However, at 3200 µM, N induced apoptosis with greater potency (*p* ≤ 0.0001) than at 8000 μM. For instance, Nisin at 3200 µM induced stronger apoptosis than at 8000 µM, likely because extreme stress at higher doses disrupts regulated apoptotic pathways and shifts cells toward necrosis or non-apoptotic death [60,61]. Very high concentrations may also reduce Nisin’s bioactivity through aggregation or excessive membrane damage [62]. Moreover, this suggests that 3200 µM achieves optimal apoptotic activation without overwhelming cellular mechanisms. UB demonstrated a dose-dependent apoptotic effect, i.e., the 500 µM concentration of UB produced a greater apoptotic induction (*p* ≤ 0.0001) compared to the untreated control, followed by 300 µM. Moreover, the N and UB combination (4:6; 3500 µM) resulted in significantly lower apoptosis cells than its monotreatments (*p* ≤ 0.05). This combination resulted in increased apoptotic cell counts (*p* ≤ 0.001) and necrotic cell populations (*p* ≤ 0.05) compared to the untreated control. Additionally, Dox (4 µM) significantly induced apoptosis as a positive control (*p* ≤ 0.0001), and its efficacy was comparable to UB at 500 µM (*p* ≤ 0.0001) compared to the untreated control, validating the assay sensitivity and providing a reference for therapeutic comparison. However, Dox treatment also induced a markedly higher proportion of necrotic cell death (*p* ≤ 0.0001) compared to the untreated control and other treatment groups.

This increase in necrosis is not considered therapeutically advantageous. Unlike apoptosis, a controlled and non-inflammatory form of cell death, necrosis results in the rupture of the cell membrane and the release of intracellular contents into the surrounding tissue [63,64], triggering local inflammation and recruiting immune cells, potentially exacerbating tissue damage and systemic side effects [64]. Clinically, this inflammatory response can contribute to cardiotoxicity, mucositis, and fatigue, all of which are commonly reported complications of Dox therapy [65]. Furthermore, these findings are consistent with emerging studies linking necroptotic signatures to chemotherapeutic overload [66]. In contrast, treatments that primarily induce apoptosis, such as N and UB in this study, are more favourable for targeted tumour clearance with reduced collateral damage. Therefore, while Dox effectively reduces tumour burden, its necrotic profile highlights the need for safer alternatives or combinatorial strategies that minimise necrosis and its associated toxicities. The synergistic induction of apoptosis observed in our study likely results from complementary mechanisms of N and UB. N, a cationic amphipathic peptide, is known to interact with anionic phospholipids in cancer cell membranes, forming pores and disrupting membrane integrity, including that of mitochondria [67,68]. This disruption leads to increased mitochondrial membrane permeability, collapse of the mitochondrial membrane potential, and consequent release of pro-apoptotic factors, such as cytochrome c, along with an elevation in intracellular reactive oxygen species (ROS). These factors together activate intrinsic (mitochondrial) apoptosis pathways [34].

In parallel, UB, a gut-microbiota-derived metabolite of ellagitannins, has been shown to impair mitochondrial oxidative phosphorylation by targeting components of the electron transport chain and modulating key metabolic pathways [40,69]. It reduces ATP production, increases oxidative stress, and promotes mitochondrial dysfunction, which triggers apoptosis through both intrinsic and extrinsic pathways. Additionally, UB can modulate signalling pathways such as NF-κB and mTOR, further sensitising cells to apoptosis [70].

In summary, UB at 500 µM induced the greatest level of apoptosis in HKB-11 lymphoma cells, significantly outperforming both the untreated control (*p* ≤ 0.0001) and N (*p* ≤ 0.01). However, the combination of N and UB in a 4:6 ratio (3200:300 µM) (*p* ≤ 0.0001) triggered a stronger apoptotic response than either compound alone, including UB at 300 µM (*p* ≤ 0.01), except for UB at 500 µM (*p* ≤ 0.0001). Therefore, this suggested a synergistic effect where the combined treatment enhances cell death more efficiently than the individual compounds. Mechanistically, UB promotes mitochondrial-driven apoptosis by modulating oxidative phosphorylation and activating pro-apoptotic signalling, while N induces membrane disruption and oxidative stress through ROS generation [71,72]. These distinct yet converging mechanisms likely account for the superior efficacy of the combination. Using N and UB together may also allow for lower individual doses, minimising toxicity while maximising therapeutic impact, highlighting the N/UB (4:6) combination as a more effective and rational approach for lymphoma treatment. The apoptosis induced by N and UB may involve the intrinsic (mitochondrial) and extrinsic (death-receptor-mediated) pathways [66]. Moreover, these findings align with contemporary research demonstrating that modulation of apoptosis through novel drug conjugates or dual-target inhibitors is pivotal in overcoming resistance in lymphoma [73,74].

### 2.5. Proteomics Study of the HKB-11 Lymphoma Cells Treated with the Synergistic Combination vs. Monotreatments

A bottom-up, label-free quantification proteomics analysis was carried out using Micro-UPLC-QTOF-MS/MS, based on a recent protocol developed by our group. The goal was to identify significant changes in the expressed proteins related to apoptosis, cancer development, cell cycle, and broad cell death functions. HKB-11 lymphoma cells were treated with N (3200 µM), UB (300 µM), and their 4:6 combination (3500 µM). Furthermore, each treatment group was compared to the control group to detect changes in the global proteome that might be associated with the antiproliferative effects. Differentially expressed proteins were selected based on strict statistical thresholds of absolute log_2_ FC ≥ 0.58 and Q ≤ 0.05. Moreover, the HKB-11 cell line was used in this experiment because it exhibits better growth characteristics, higher transfection efficiency, and a greater protein yield, making it more suitable for proteomic studies. These technical advantages are critical for mass-spectrometry-based analyses and pathway enrichment [5].

#### 2.5.1. Differentially Expressed Proteins (DEPs) in N (3200 µM)-Treated HKB-11 Lymphoma Cells Compared to Untreated Control (Abs Log_2_FC ≥ 0.58 and Q ≤ 0.05)

**Figure 3:**
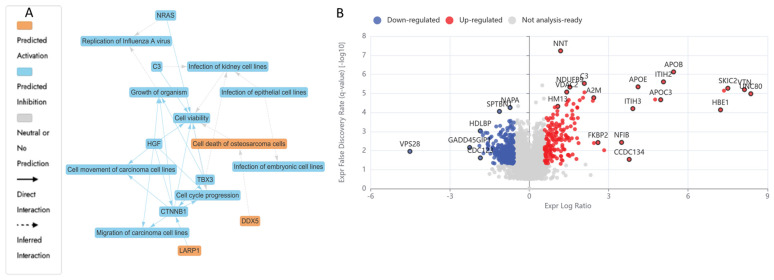
Proteomic response of HKB-11 lymphoma cells to Nisin treatment. Cells were treated with Nisin (N; 3200 µM) for 24 h, and protein expression was analysed using label-free quantitative proteomics. (**A**) Ingenuity Pathway Analysis (IPA) graphical summary highlighting the top predicted biological functions affected by Nisin treatment. The analysis indicates significant inhibition of cell cycle progression and cell movement. (**B**) Volcano plot displaying the distribution of all quantified proteins. The x-axis represents the log_2_ fold change (Log_2_FC) in protein expression, and the y-axis represents the statistical significance (−log_10_ Q-value). Proteins that were significantly upregulated (Log_2_FC ≥ 0.58, Q ≤ 0.05) are shown in red, and those significantly downregulated (Log_2_FC ≤ −0.58, Q ≤ 0.05) are shown in blue. Nisin treatment predominantly downregulated proteins involved in cell proliferation and survival.

LCMS-based bottom-up proteomic profiling of HKB-11 lymphoma cell lysate after treatment with N at 3200 µM is presented in Table S1, with the Ingenuity Pathway Analysis (IPA) enrichment report listed in Table S2 (Supplementary Materials). Moreover, Table 4 lists the anticancer-related proteins dysregulated upon N treatment. Furthermore, Figure 3A shows the IPA graphical summary, along with the volcano plot in Figure 3B of N compared to the untreated control cell lysate.

The IPA graphical summary in Figure 3A offers a concise visualisation of the key biological insights derived from the IPA Core Analysis. It highlights and links a selected subset of the most statistically relevant entities, such as upstream regulators and biological functions, into an integrated overview. By leveraging machine learning, the system prioritises these entities and infers potential relationships, even when direct connections are not yet documented in the QIAGEN Knowledge Graph. These inferred links help depict the broader biological context and interactions uncovered in the analysis.

IPA summary upon using the DEPs of N vs. the untreated control revealed several key biological themes central to the network that emerged as a dominant concept, influenced by regulators such as *C3*, *CTNNB1*, *HGF*, *NRAS*, and *TBX3*, all of which contribute to maintaining or disrupting cellular survival [75]. The inhibition of cell cycle progression is closely linked and regulated by similar factors, including *CTNNB1* and *HGF*, indicating their role in governing cell division and growth processes essential for proper cellular and organismal development [75,76]. Cell migration and motility also play a critical role in the network, with *CTNNB1* and *HGF* significantly impacting carcinoma cell movement, thereby connecting this theme to processes such as tissue repair and cancer metastasis. Lastly, organismal growth is strongly affected by *CTNNB1*, *HGF*, and *TBX3*, pointing to their influence on developmental biology [75,76,77]. The N treatment also inhibited several vital proliferative and survival pathways in the HKB-11 cells. Proteins essential for nucleotide synthesis, DNA replication, and stress response, such as TYMS (Log_2_FC = −1.54), LARP1 (Log_2_FC = −1.27), MAPK14 (Log_2_FC = −0.80), SRPK2 (Log_2_FC = −0.70), CDK4 (Log_2_FC = −1.26), PRC1 (Log_2_FC = −0.72), RFC1 (Log_2_FC = −0.85), RFC2 (Log_2_FC = −0.59), and PCNA (Log_2_FC = −0.63), were notably downregulated. The reduced expression of *TYMS* directly inhibits DNA synthesis, thereby impairing cell proliferation [78,79]. Likewise, diminished *LARP1* and *MAPK14* levels indicated potential suppression of mTOR-mediated translation and stress-responsive mitogenic signalling pathways, crucial for cancer cell survival [80,81]. Furthermore, downregulation of *CDK4*, *PRC1*, *RFC1*, *RFC2*, and *PCNA* was previously linked to a significant inhibition of cell cycle progression, particularly halting the G_1_/S transition and DNA synthesis phase, resulting in cell cycle arrest and apoptosis [82,83,84]. Moreover, the decreased HDAC1 expression (Log_2_FC = −0.67) observed in this study may contribute to tumour inhibition through epigenetic modulation, enhancing susceptibility to apoptosis [85].

Figure 3 illustrates enriched pathways (IPA, Q ≤ 0.05, absolute z-score ≥ 2 or ≤−2) linked explicitly to cell adhesion, cancer proliferation, and mRNA processing. The altered expression profiles suggested a dual impact of N treatments, suppressing key cell cycle and DNA replication pathways while disrupting metabolic processes essential for tumour survival. Additionally, proteins such as *CTNNB1*, *HGF*, and *TBX3*, which are involved in cell viability, movement, migration, and organismal growth, indicate broader implications of genetic modulation on developmental biology and disease dynamics, as the same genes that control development often drive disease when dysregulated. However, mutations or altered expression can lead to cancer, fibrosis, or developmental disorders [86,87,88]. In summary, these findings depict a complex interplay induced by N treatment, balancing proliferative adaptations with robust inhibitory mechanisms, ultimately influencing lymphoma cell viability and tumour progression pathways.

**Table 4:** Key differentially expressed proteins (DEPs) involved in anticancer mechanisms following treatment with Nisin, Urolithin B, or their combination. HKB-11 lymphoma cells were treated for 24 h with Nisin (N; 3200 µM), and Urolithin B (UB; 300 µM) compared to untreated control cells or the N/UB 4:6 combination (3200:300 µM) vs. monotreatments. Q ≤ 0.05 and absolute log_2_ fold change (Log_2_FC) ≥ 0.58 were applied, where blue and red colours were used to indicate downregulation and upregulation, respectively. The table summarises the associated molecular pathway, mechanism of action, and relevant references for each protein, highlighting effects on cell cycle, apoptosis, metabolism, and immune response.

Treatment	Log_2_FC	*Gene* ID	Protein Descriptions	Molecular Pathway	Mechanism of Action	Reference
N 3200 μM	−1.27	*LARP1*	La-related protein 1	mTOR signalling and RNA binding	Regulates mRNA stability and translation of survival genes	[80,89]
	−1.54	*TYMS*	Thymidylate synthase	Nucleotide synthesis and DNA replication	Catalyses thymidylate synthesis; target of 5-FU chemotherapy	[78,79]
	−0.80	*MAPK14*	Mitogen-activated protein kinase 14	MAPK/p38 signalling pathway	Mediates cellular response to stress, inflammation, and proliferation	[81]
	−0.72	*PRC1*	Protein regulator of cytokinesis 1	Cell cycle progression and mitosis	Regulates cytokinesis and mitotic spindle formation	[90]
	−0.70	*SRPK2*	SRSF protein kinase 2	RNA splicing and nuclear mRNA processing	Works with SRPK1 in regulating alternative splicing	[91,92]
	−1.26	*CDK4*	Cyclin-dependent kinase 4	Cell cycle (G1/S transition)	Phosphorylates RB1, promoting E2F release and progression through G1 phase	[82]
	−0.62	*POLR2E*	DNA-directed RNA polymerases I, II, and III subunit RPABC1	Transcription (RNA Polymerase II complex)	Essential subunit for RNA Polymerase II assembly and mRNA transcription	[93]
	−0.63	*POLR2G*	DNA-directed RNA Polymerase II subunit RPB7	Transcription (RNA Polymerase II complex)	Structural component maintaining Polymerase II processivity	[94,95]
	−0.59	*POLR2H*	DNA-directed RNA polymerases I, II, and III subunit RPABC3	Transcription (RNA Polymerase II complex)	Stabilises RNA Pol II structure; shared across all RNA polymerases	[95,96]
	−0.85	*RFC1*	Replication factor C subunit 1	DNA replication (clamp loader complex)	Loads PCNA onto DNA, facilitating DNA polymerase binding during replication	[97]
	−0.59	*RFC2*	Replication factor C subunit 2	DNA replication and repair	Binds RFC1 to form RFC complex; essential for DNA synthesis fidelity	[83]
	−0.60	*NUP62*	Nuclear pore glycoprotein p62	Nucleocytoplasmic transport (nuclear pore complex)	Central channel component; regulates import/export of macromolecules	[98]
	−0.93	*PPP1R14B*	Protein phosphatase 1 regulatory subunit 14B	Actin cytoskeleton regulation	Inhibits protein phosphatase 1, affecting the cytoskeleton and cell motility	[99,100]
	−0.97	*CTNNA1*	Catenin alpha-1	Cell adhesion (cadherin complex)	Links cadherins to actin cytoskeleton; maintains epithelial integrity	[101]
	−0.67	*HDAC1*	Histone deacetylase 1	Epigenetic regulation (histone deacetylation)	Removes acetyl groups from histones, repressing transcription	[85]
UB 300 μM	2.04	*APOC3*	Apolipoprotein C-III	Triglyceride metabolism	Alters lipid signalling; enhances inflammatory microenvironment	[102]
	4.39	*C4BPA*	C4b-binding protein alpha chain	Complement pathway	Inhibits complement-mediated lysis; immune evasion	[103]
	3.06	*CLEC11A*	C-type lectin domain family 11 member A	Cytokine signalling	Promotes endothelial and haematopoietic support in TME	[104]
	2.07	*COX6C*	Cytochrome c oxidase subunit 6C	Respiratory chain complex IV	Boosts mitochondrial respiration	[105]
	2.08	*COX7C*	Cytochrome c oxidase subunit 7C, mitochondrial	Cytochrome c oxidase	Increases mitochondrial adaptability in tumours	[106]
	2.14	*FGB*	Fibrinogen beta chain	Coagulation cascade	Promotes vascularisation and fibrin scaffolding in tumours	[107]
	2.26	*GSN*	Gelsolin	Actin regulation	Modulates the actin cytoskeleton for migration and invasion	[108]
	5.94	*HBD*	Haemoglobin subunit delta; Haemoglobin subunit beta	Haemoglobin complex	Facilitates oxygen delivery; modulates redox status	[109]
	5.63	*HBE1*	Haemoglobin subunit epsilon; Haemoglobin subunit gam1; Haemoglobin subunit gamma-2	Foetal haemoglobin pathway	Reactivation may aid hypoxic survival in tumours	[110]
	2.76	*ITIH2*	Inter-alpha-trypsin inhibitor heavy chain H2	Matrix stability	Regulates hyaluronic acid and ECM stiffness	[111]
	2.34	*MT-CO2*	Cytochrome c oxidase subunit 2	Mitochondrial respiration	Supports tumour ATP production and ROS balance	[112]
	2.24	*MT-ND4*	NADH-ubiquinone oxidoreductase chain 4	Complex I, OXPHOS	Enhances mitochondrial respiration and survival under stress	[113]
	2.18	*NDUFA11*	NADH dehydrogenase [ubiquinone] 1 alpha subcomplex subunit 11	OXPHOS complex I	Maintains mitochondrial metabolism in cancer cells	[114]
	2.01	*SAMM50*	Sorting and assembly machinery component 50 homolog	Protein import	Preserves outer membrane; supports anti-apoptotic signals	[115]
	−1.45	*MAD2L1*	Mitotic spindle assembly checkpoint protein MAD2A	Mitotic checkpoint (spindle assembly checkpoint, SAC)	Ensures proper chromosome segregation, disrupts mitosis, causing mitotic arrest or apoptotic cell death	[116]
	−0.59	*PSME3*	Proteasome activator complex subunit 3	Proteasome activation, p53 degradation	Degrades tumour suppressor proteins. Its suppression stabilises p53, enhancing apoptosis and cell cycle arrest	[117]
	−1.21	*UBE2S*	Ubiquitin-conjugating enzyme E2 S	Ubiquitination, mitotic exit	Ubiquitinates mitotic inhibitors (APC/C complex co-activator); downregulation leads to mitotic arrest, promoting senescence	[118]
	−0.63	*PCNA*	Proliferating cell nuclear antigen	DNA replication and repair	Sliding clamp for DNA polymerases; loss of PCNA function causes replication stress and apoptosis	[84]
	−1.69	*UBE2E1*	Ubiquitin-conjugating enzyme E2 E1; Ubiquitin-conjugating enzyme E2 E3; Ubiquitin-conjugating enzyme E2 E2	Ubiquitin-conjugating enzyme	Supports proteostasis, DNA repair; suppression disrupts protein quality control, leading to cell death	[119]
N/UB 4:6 (3200:300 μM)	2.30	*A2M*	Alpha-2-macroglobulin	Protease inhibition, complement cascade	Regulates proteolysis; potentially restricts tumour invasion	[120]
	0.67	*CDC27*	Cell division cycle protein 27 homolog	Mitotic checkpoint (Anaphase-Promoting Complex, APC/C)	Regulates ubiquitination of mitotic regulators	[121]
	2.45	*AFP*	Alpha-fetoprotein	Oncofoetal protein, MAPK signalling	Supports tumour proliferation, angiogenesis; marker in hepatic and haematological cancers	[122]
	2.56	*AHSG*	Alpha-2-HS-glycoprotein	TGF-β inhibition	Inhibits calcification and regulates inflammation in tumours	[123]
	2.32	*ALB*	Albumin	Plasma transport	High levels may reflect cancer cachexia or liver activity during tumour burden	[124]
	6.55	*ALG6*	Dolichyl pyrophosphate Man9GlcNAc2 alpha-1,3-glucosyltransferase	N-glycosylation	Promotes ER glycoprotein processing; linked to tumour cell survival	[125]
	2.64	*IGLL5*	Immunoglobulin lambda-like polypeptide 5	B-cell development	Overexpressed in some B-cell lymphomas; immune receptor surrogate	[126]
	2.36	*LCAT*	Phosphatidylcholine-sterol acyltransferase	HDL metabolism	Alters cholesterol availability in tumours	[127]
	2.46	*NNMT*	Nicotinamide N-methyltransferase	Nicotinamide methylation	Reprograms NAD+ metabolism; supports proliferation	[128]
	2.38	*SERPINA7*	Thyroxine-binding globulin	Thyroid hormone transport	Affects hormone signalling relevant to cancer cell growth	[129]
	2.27	*SERPINF1*	Pigment epithelium-derived factor	Anti-angiogenic	Inhibits neovascularisation; tumour-suppressive in some contexts	[130]
	3.41	*TF*	Serotransferrin	Iron transport	Modulates iron availability and oxidative stress in tumour cells	[131]
	1.78	*F10*	Coagulation factor X	Coagulation cascade	Activation of prothrombin to thrombin; can affect tumour vascularization	[132]
	0.71	*FGB*	Fibrinogen beta chain	Extracellular matrix (ECM) interaction	Participates in clot formation and tissue remodelling	[107]
	0.65	*ITGB1*	Integrin beta-1	Cell adhesion, survival signalling	Binds ECM; activates FAK, PI3K pathways	[133]
	0.77	*RANGAP1*	Ran GTPase-activating protein 1	Nuclear transport and cell cycle regulation	Controls Ran GTPase cycle; vital for nuclear envelope reformation during mitosis	[134]
	−0.75	*BUB3*	Mitotic checkpoint protein BUB3	Spindle assembly checkpoint (SAC)	Ensures correct chromosomal segregation; loss promotes chromosomal instability but can also trigger catastrophic cell death in tumours	[135]
	−1.18	*CCNB1*	G2/mitotic-specific cyclin-B1	Cell cycle control (G2/M checkpoint)	Complexes with CDK1 to trigger mitosis; downregulation leads to G2/M arrest and apoptosis	[136]
	−0.60	*CDCA8*	Borealin	Chromosome passenger complex (CPC)	Regulates mitosis and cytokinesis; loss disrupts chromosomal stability, causing mitotic catastrophe	[137]
	−0.66	*CDK1*	Cyclin-dependent kinase 1	Master G2/M checkpoint kinase	Phosphorylates downstream mitotic proteins; inhibition causes G2/M phase arrest, senescence, or apoptosis	[138]
	−0.59	*MAP2K4*	Dual-specificity mitogen-activated protein kinase kinase 4	JNK/p38 MAPK stress pathway	Activates pro-apoptotic MAPK cascades; blocks survival signals, sensitizing cells to apoptosis	[139,140]

#### 2.5.2. Differentially Expressed Proteins (DEPs) in UB (300 µM)-Treated HKB-11 Lymphoma Cells Compared to Untreated Control (Abs Log_2_FC ≥ 0.58 and Q ≤ 0.05)

**Figure 4:**
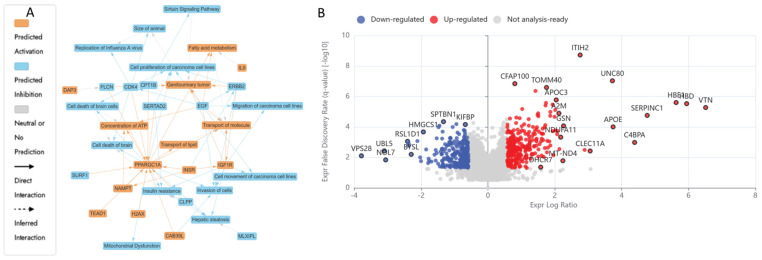
Proteomic response of HKB-11 lymphoma cells to Urolithin B treatment. Cells were treated with Urolithin B (UB; 300 µM) for 24 h, and protein expression was analysed using label-free quantitative proteomics. (**A**) Ingenuity Pathway Analysis (IPA) graphical summary illustrating the top predicted biological functions affected by UB treatment, highlighting pathways related to mitochondrial function, lipid metabolism, and cancer cell proliferation. (**B**) Volcano plot showing protein expression changes. The x-axis is the log_2_ fold change (Log_2_FC), and the y-axis is the statistical significance (− log_10_ Q-value). Significantly upregulated proteins (Log_2_FC ≥ 0.58, Q ≤ 0.05) are in red, and significantly downregulated proteins (Log_2_FC ≤ − 0.58, Q ≤ 0.05) are in blue. UB treatment robustly upregulated proteins of the mitochondrial respiratory chain while downregulating key mitotic regulators, thereby inhibiting the invasion, proliferation, and metastasis of cancer cells.

The IPA graphical summary in Figure 4B highlights several biological themes enriched by significantly regulated proteins (Q ≤ 0.05, Abs Log_2_FC ≥ 0.58) upon UB treatment. One major theme is metabolic regulation and mitochondrial function, which suggests significant regulation of metabolic pathways and mitochondrial activities, particularly via *PPARGC1A* (PGC-1α). This component plays a central role in fatty acid metabolism, molecule transport, and mitigating mitochondrial dysfunction, positioning it as a key regulator of mitochondrial biogenesis and energy metabolism [141]. Another important theme is the proliferation and invasion of cancer cells, where proteins such as *CDK4*, *EGF*, and *ERBB2* play a crucial role in controlling the growth and metastasis of carcinoma cell lines. This theme is crucial for understanding the mechanisms of cancer progression and identifying potential therapeutic targets. The theme of insulin resistance and hepatic steatosis was also prominent, with genes such as *CLPP*, *IGF1R*, and *PPARGC1A* influencing metabolic disorders like diabetes and non-alcoholic fatty liver disease (NAFLD), indicating potential pathways for intervention [142]. Lastly, the theme of genitourinary tumours is highlighted, where *ERBB2* and *IGF1R* are involved in pathways leading to the development and progression of genitourinary cancers. This theme offers insights into cancers affecting the urinary and reproductive systems and explores potential therapeutic options.

Comprehensive proteomic profiling of HKB-11 lymphoma cells after treatment with UB at 300 µM is presented in the attached Supplementary Materials, with Tables S1 and S3 being summarised in Table S5 and visualised in Figure 4A,B. Treatment with UB significantly enhanced mitochondrial activity in lymphoma cells, prominently upregulating key components of the mitochondrial respiratory chain, including COX6C (Log_2_FC = 2.07), COX7C (Log_2_FC = 2.08), MT-CO2 (Log_2_FC = 2.34), MT-ND4 (Log_2_FC = 2.24), and NDUFA11 (Log_2_FC = 2.01), as illustrated in Figure 4. This mitochondrial reprogramming was underscored by enhanced oxidative phosphorylation, which is essential for ATP production and cellular survival under oxidative stress conditions commonly present in tumour microenvironments [112,113]. In contrast, treatment with UB also induced notable downregulation of critical proteins involved in mitotic progression and proteostasis, such as MAD2L1 (Log_2_FC = −1.45), UBE2S (Log_2_FC = −1.21), and UBE2E1 (Log_2_FC = −1.69). However, these proteins are pivotal in ensuring accurate cell cycle progression and genomic stability. Their suppression indicated disruption of mitotic control mechanisms, leading to mitotic arrest, increased genomic instability, and apoptosis [116,118,119]. Thus, UB treatment reveals a dual action mechanism that simultaneously enhances mitochondrial metabolic resilience and impairs cell division pathways, collectively influencing lymphoma cell fate.

#### 2.5.3. Differentially Expressed Proteins (DEPs) in Combo N/UB (4:6)-Treated HBK-11 Cells vs. Monotreatments (N 3200 µM and UB 300 µM) (Abs Log_2_FC ≥ 0.58 and Q ≤ 0.05)

**Figure 5:**
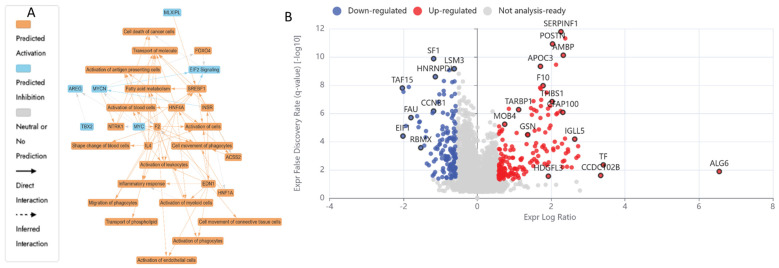
Synergistic proteomic changes in HKB-11 cells treated with the Nisin and Urolithin B combination. This analysis compares the proteome of cells treated with the N/UB 4:6 combination (3200:300 µM) for 24 h against cells treated with Nisin or Urolithin B alone to spot the synergistic mechanisms. (**A**) Ingenuity Pathway Analysis (IPA) graphical summary of biological functions uniquely enriched by the combination treatment, showing strong modulation of immune response, cell movement, and metabolism. (**B**) Volcano plot illustrating the differentially expressed proteins in the combination treatment versus monotherapies. The x-axis is the log_2_ fold change (Log_2_FC), and the y-axis is the statistical significance (− log_10_ Q-value). Significantly upregulated proteins (Log_2_FC ≥ 0.58, Q ≤ 0.05) are in red, and significantly downregulated proteins (Log_2_FC ≤ − 0.58, Q ≤ 0.05) are in blue. The combination therapy elicited a distinct and more potent proteomic response via activating cell death.

The IPA graphical summary Figure 5B reveals key biological themes enriched within the gene network, based on significantly regulated proteins (Q ≤ 0.05, Abs Log_2_FC ≥ 0.58). A central theme is the inflammatory response, prominently influenced by genes such as *EDN1*, *F2*, and *HNF4A*, which drive increased inflammation by activating pathways associated with myeloid cells and immune signalling [143,144]. Closely related is the activation of immune cells, with *EDN1*, *INSR*, *IL4*, and others playing roles in stimulating antigen-presenting cells, leukocytes, and phagocytes, highlighting immune activation as a core network component. Another key theme is fatty acid metabolism, governed by the actions of *EDN1*, *F2*, and *SREBF1*, underscoring the relevance of lipid processing and energy utilisation in the system [145,146,147]. Cell movement, especially that of phagocytes, also emerges as a significant motif, facilitated by *ACSS2*, *EDN1*, and *IL4*, which are critical for immune surveillance and tissue remodelling [148,149]. Lastly, the transport of molecules is a recurring and interconnected theme, with involvement from *F2*, *HNF4A*, *INSR*, *IL4*, *NTRK1*, and *SREBF1*, indicating the pivotal role of intracellular and intercellular transport in maintaining functional homeostasis within the biological network [150].

Comprehensive proteomic profiling of HKB-11 lymphoma cells after treatment with N/UB (4:6), N 3200 µM, and UB 300 µM is presented in the attached Supplementary Materials, with Tables S1 and S4 being summarised in Table S5 and visualised in Figure 5A,B. Combination therapy using N and UB (4:6) intensified anticancer effects via multiple complementary mechanisms. Notably, proteins crucial to metabolic reprogramming were significantly upregulated, including NNMT (Log_2_FC = 2.46), which alters NAD+ metabolism, potentially restricting tumour growth through metabolic modulation (Sun et al., 2024) [128]. Additionally, proteins involved in immune modulation and tumour microenvironment remodelling, such as A2M (Log_2_FC = 2.30) and SERPINF1 (Log_2_FC = 2.27), were also elevated, suggesting a potential reduction in tumour invasiveness and angiogenesis [120,130]. Conversely, key regulators of cell cycle progression and chromosome segregation, BUB3 (Log_2_FC = −0.75), CCNB1 (Log_2_FC = −1.18), CDCA8 (Log_2_FC = −0.60), and CDK1 (Log_2_FC = −0.66), were markedly downregulated. This suppression potentially indicated disrupted mitotic progression, genomic instability, and potential mitotic catastrophe, ultimately arresting tumour proliferation [135,136,137,138].

**Figure 6:**
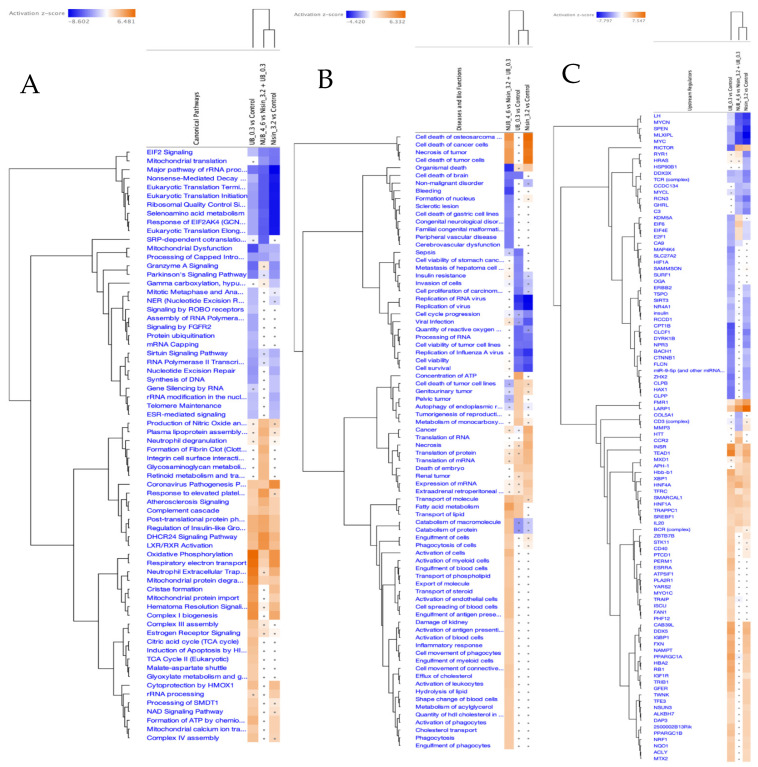
Comparative pathway analysis of Nisin, Urolithin B, and combination treatments in HKB-11 cells. Ingenuity Pathway Analysis (IPA) was used to compare the effects of Nisin (3200 µM) and Urolithin B (300 µM) against an untreated control, along with their 4:6 combination (N/UB) vs. the monotreatments. Heatmaps display the activation z-score, where orange colour indicates predicted pathway activation and blue indicates predicted inhibition (filtered by Q ≤ 0.001 and |z-score| ≥ 2). (**A**) Canonical pathways analysis shows the synergistic pathways related to combination therapy, where the monotreatment vs. Ctr outlines the enriched pathway related to its cytotoxic effect in HBK-11 cells. (**B**) Disease and bio-function analysis reveals the combination therapy has a stronger predicted inhibitory effect on non-malignant disorder, organismal death, and cell death, while enhancing the activation of fatty acid metabolism and phagocytosis. (**C**) Upstream regulator analysis predicts stronger inhibition of key oncogenic regulators like MYC, MYCN, and E2F1 by the combination treatment compared to monotherapies.

Comparative pathway analysis demonstrated enhanced effects unique to the combination treatment. Figure 6A reveals significantly altered canonical pathways, notably those governing cell cycle regulation, oxidative stress response, and immune-related signalling such as IL-6, TREM1, and NF-κB. Figure 6B presents enriched disease and biological functions, emphasising the suppression of tumour proliferation, reduced metastatic potential, and heightened activation of leukocyte migration and immune cell responses. Figure 6C presents the predicted upstream regulators modulated by the combination. Notably, MYC, MYCN, and SPEN are predicted to be inhibited, consistent with the suppression of oncogenic drivers. This pattern suggests coordinated downregulation of pathways supporting proliferation and survival, reinforcing the observed synergistic impact on oncogenic and immunological signalling networks. Collectively, these proteomic findings highlighted a nuanced interplay among metabolic adjustments, mitochondrial flexibility, immune regulation, and cell cycle inhibition. Thus, the data robustly supported the therapeutic efficacy of N and UB, individually and synergistically, in lymphoma management by targeting pivotal cellular pathways essential for tumour cell survival and proliferation.

## 3. Materials and Methods

### 3.1. Chemicals and Drug Preparation

All the metabolites used in the study, N and UB, were purchased from Sapphire Bioscience (Redfern, NSW, Australia). Magnesium acetate (MgA), sodium propionate (NaP), sodium butyrate (NaB), and doxorubicin (Dox) were also purchased from Sigma Aldrich (Castle Hill, NSW, Australia). Furthermore, all reagents were prepared according to the standard methods and protocols provided with the assay kits.

### 3.2. Cell Culture

Hs 313.T (ATCC CRL-7235) passage (4), HKB-11 (ATCC CRL-12568; human kidney/B cell Hybrid) passage (4–10), and HS-5 (ATCC CRL-3611) passage (5) were purchased from the American Type Culture Collection (ATCC, Manassas, VA, USA). Hs 313.T lymphoma cells were grown in ATCC-formulated Dulbecco’s Modified Eagle’s Medium (DMEM; ATCC 30-2002), comprising of 4.5 g/L glucose, L-glutamine, and sodium pyruvate supplemented with 10% foetal bovine serum (FBS; Bio-Strategy PTY, Campbellfield, VIC, Australia), supplemented with 1% penicillin and streptomycin (Sigma Aldrich, Castle Hill, NSW, Australia). The HKB-11 cells were grown in the ATCC-formulated DMEM/F12 (1:1 mixture of DMEM and Ham’s F-12) supplemented with 10% FBS (Bio-Strategy PTY, Campbellfield, VIC, Australia) and 1% penicillin and streptomycin (Sigma Aldrich, Castle Hill, NSW, Australia). HS-5 normal cells were grown in the ATCC-formulated DMEM (ATCC 30-2002), comprising 4.5 g/L glucose, L-glutamine, and sodium pyruvate, supplemented with 10% FBS (Bio-Strategy PTY, Campbellfield, VIC, Australia) and 1% penicillin and streptomycin (Sigma Aldrich, Castle Hill, NSW, Australia). These cells were maintained at 37 °C in a 5% controlled CO_2_ atmosphere, and cell maintenance was performed every 48–72 h, which is the time necessary for cells to achieve confluent monolayers.

### 3.3. Cell Viability Assays

The cell viability of the HKB-11, Hs 313.T, and HS-5 cells after treatment with different concentrations of the seven different postbiotics, including SCFA salts (magnesium acetate, sodium propionate, and sodium butyrate), UA and UB, purine nucleoside (inosine), and bacteriocin (N), was determined using the Alamar Blue assay as per the method described earlier [151,152]. Briefly, 100 μL of cells was cultured in 96-well plates at a 3 × 10^5^ cells/mL seeding density. After 24 h, the cells were treated with 16,000 µM of each postbiotic except for the urolithins, which were applied at 500 µM using a 1:2 serial dilution across a 10-point dose–response curve, followed by a 72 h incubation. A positive control using doxorubicin was prepared at a concentration of 4 μM, and an untreated control with 0.1% DMSO was added to every plate. At the end of the incubation period, the culture media were removed, and 100 μL of a 0.1 mg/mL Alamar Blue solution (resazurin, prepared as a stock solution at 1 mg/mL in freshly made PBS, followed by a 1:10 dilution with serum-free media) was added to each well. The fluorescence levels were assessed using a microplate spectrophotometer (BMG CLARIOstar, Mornington, VIC, Australia) with an excitation wavelength of 555 nm and emission measurement at 595 nm. The compounds were tested in triplicate, with the untreated control taken as 100% cell viability.

### 3.4. Synergy Analysis

The most potent metabolites (N and UB) were combined at nine different ratios, 1:9 *v*/*v* (800:450 μM), 2:8 *v*/*v* (1600:400 μM), 3:7 *v*/*v* (2400:350 μM), 4:6 *v*/*v* (3200:300 μM), 5:5 *v*/*v* (4000:250 μM), 6:4 *v*/*v* (4800:200 μM), 7:3 *v*/*v* (5600:150 μM), 8:2 *v*/*v* (6400:100 μM), and 9:1 *v*/*v* (7200:50 μM), with a 1:2 serial dilution ratio, for combination index (CI) analyses. This study used the CI model to show the interaction between N and UB. CompuSyn version 2.0 (Biosoft, San Francisco, CA, USA) was used for our calculations. Moreover, this software calculates CI based on the median-effect equation from the mass action law [151]. The current study used the CI model to study the nine pairwise postbiotic combinations with a six-point dose–response curve. The CI model quantifies the potential interactions between drug–drug combinations into three categories: (a) synergistic effect: CI value ≤ 1, (b) additive effect: CI = 1, and (c) antagonistic effect: CI value ≥1.

### 3.5. Analysis of ROS Production

The effect of the most active gut metabolites and their most synergistic combinations on the oxidative stress of the lymphoma cells was studied as per the protocol using the H2DCFDA (2′,7′-dichlorofluorescein diacetate) cellular ROS Detection Assay Kit (#ab113851; Abcam, Melbourne, VIC, Australia) [25,152]. Briefly, HKB-11 lymphoma cells (2.5 × 10^5^ cells/mL) were cultured in a 96-well plate, allowed to adhere overnight, and treated with 20 μM H2DCFDA for 45 min to assess ROS levels. The dye solution was removed, and cells were washed with 1× buffer. Next, the cells were treated with N (8000 μM), N (4000 μM), UB (500 μM), UB (250 μM), N/UB (3500 μM), N/UB (1750 μM), N/UB (5750 μM), N/UB (2875 μM), Dox (4 μM), and tert-butyl hydroperoxide (TBHP) (150 μM) and then incubated at 37 °C for 4 h. Finally, the plate was immediately read at Ex/Em = 485/535 nm using a microplate spectrophotometer (BMG CLARIOstar, VIC, Australia). The fold change in ROS production was determined relative to the untreated control (cells treated with the supplement buffer according to the manufacturer’s protocol).

### 3.6. Flow Cytometry Analyses of the Apoptotic Profiles

The impact of the most potent postbiotics and their most synergistic combinations on the apoptosis profiles of the HKB-11 lymphoma cells after 24 h treatment was studied using an Annexin V- and 7-AAD-based kit (#ab214663, Abcam, Melbourne, VIC, Australia) [25,152]. The HKB-11 cells were cultured in T75 cell culture flasks with an initial density of 1 × 10^6^ cells per 10 mL at 37 °C in the presence of 5% CO_2_ for 24 h. The following day, the cell culture media was removed from each flask and replaced with fresh FBS-containing media. The cultured flasks were then treated with the highest concentration of the most active postbiotics N (8000 μM) and UB (500 μM) and the positive control Dox (4 μM). An FBS-containing medium was used as the untreated control. The flasks were then incubated at 37 °C with 5% CO_2_ for 24 h. Then, the cell culture media from each flask were collected. Subsequently, trypsin (0.25% *w*/*v*) was applied to the flasks for 4 min at 37 °C. The trypsin reaction was neutralised with an equal volume of 10% FBS serum-containing media, and the cells were combined with the previously collected media. The cell pellets were obtained by centrifuging at 500× *g* for 5 min at room temperature (RT). This procedure was repeated by suspending the cell pellets in 1 mL of PBS each time. The collected cell pellets from each treatment were immediately suspended in 500 μL of 1× binding buffer and gently mixed by pipetting. Annexin V-CF Blue (5 μL) and 7-AAD (5 μL) staining solutions were added to 100 μL of the cell suspension. The stained cells were incubated for 15 min in the dark at RT, after which 400 μL of a 1× assay buffer was added to each cell suspension. Subsequently, the cells were examined using a flow cytometer (Novocyte 3000, ACEA Biosciences Inc., San Diego, CA, USA), and data analysis and processing were performed using NovoExpress software (version 1.5.0, ACEA Biosciences Inc., CA, USA). In the initial step, the cells were gated on forward and side scatter modes to exclude cell aggregates and debris near the origin. The cells were then gated on dot plots, where Annexin V-CF in Pacific Blue was plotted against 7-AAD fluorescence in PerCP. Quadrants were positioned relative to the untreated control, indicating live cells (+Annexin V and −7-AAD) appearing in the lower-left quadrant, early apoptotic cells (+Annexin V and −7-AAD) in the lower-right quadrant, late apoptotic cells (+Annexin V and +7-AAD) in the upper-right quadrant, and necrotic cells (−Annexin V and +7-AAD) in the upper-left quadrant. For statistical analyses and visualisation, the percentage data of cells in each quadrant after different treatments (n = 3) were exported to GraphPad Prism software (version 9.0, San Diego, CA, USA).

### 3.7. Liquid Chromatography–Mass Spectrometry (LC–MS)-Driven Bottom-Up Proteomics Analysis

#### 3.7.1. Cell Culture, Treatment, and Protein Extraction

The HKB-11 lymphoma cells were placed in 6-well plates at a density of 3.0 × 10^6^ cells/well and incubated overnight at 37 °C in 5% CO_2_. After the media were removed, they were replaced with fresh DMEM/F-12 medium supplemented with 10% FBS, and the cultured flasks were treated with specific doses of the most active postbiotics and their combinations. Treatments were performed in triplicate and incubated for 24 h under the same conditions. Following incubation, each flask of cells was subjected to a 0.25% *w*/*v* trypsin treatment for 4 min at 37 °C, and the cell culture medium was collected. Additionally, an equal volume of DMEM F-12 medium (containing 10% FBS) was added before mixing with the previously collected media to neutralise the trypsin. The cells were spun in a centrifuge at 500× *g* for 5 min at RT. The cell pellets were washed twice with ice-cold PBS and spun again at 500× *g* for 5 min. These cell pellets were then suspended in a lysis buffer that included 1 μL of universal nuclease (Easypep Mini Kit) (Thermo Fisher Scientific, Sydney, NSW, Australia) and supplemented with Halt™ Protease and Phosphatase Inhibitor Cocktail in a 1:100 ratio (Thermo Fisher Scientific, Sydney, NSW, Australia). The cells were gently pipetted 10–15 times to reduce the sample’s viscosity and then placed on ice for 20 min. The lysate was centrifuged at 14,000 rpm for 20 min at 4 °C, and the resulting liquid was collected.

#### 3.7.2. Protein Quantification

The Pierce™ Rapid Gold BCA Protein Assay Kit (#A53226, Thermo Fisher Scientific, Sydney, NSW, Australia) was used to determine the protein concentration of the cell lysate in triplicate, using a bovine serum albumin (BSA) standard, following the manufacturer’s protocol [24,25]. In brief, 1 μL of each sample replicate was diluted 1:20 in Milli-Q water, along with 20 μL of each standard, and then placed in a 96-well plate with 200 μL of working reagent in each well. Samples were diluted to a concentration within the 20–2000 μg/mL working range. The plate was thoroughly mixed on a plate shaker for 30 s and incubated at RT for 5 min, and then the absorbance was measured within 20 min at 480 nm using a microplate spectrophotometer (BMG CLARIOstar, Melbourne, VIC, Australia). The blank absorbance was subtracted from all other readings of standards and samples, and the sample concentration was determined using the established BSA standard calibration curve. The samples were then stored at −80 °C for further analysis.

#### 3.7.3. Peptide Preparation and Clean-Up

The protein samples (100 μg) were subjected to chemical and enzymatic sample processing using the EasyPep™ Mini MS Sample Prep Kit following the manufacturer’s instructions (Thermo Fisher Scientific, Sydney, NSW, Australia) and as reported in the literature [24,25,153]. Briefly, the sample volume was adjusted to 100 μL using a lysis buffer in a microcentrifuge tube. Subsequently, the reduction and alkylation solutions (50 μL each) were introduced, gently mixed, and incubated at 95 °C with a heat block for 10 min. The samples were allowed to cool to RT, after which 50 μL of the reconstituted trypsin/lys-C protease mixture was added to each sample and incubated with shaking at 37 °C for 3 h. Following incubation, 50 μL of a digestion stop solution was gently mixed into the samples. Peptide clean-up columns were used to remove both hydrophilic and hydrophobic impurities. The resulting clean peptide samples were dehydrated using a vacuum centrifuge and reconstituted in 100 μL of a 0.1% formic acid solution in water for LC–MS analysis. Subsequently, these samples were carefully transferred to maximum recovery sample vials (Waters Corp., Milford, MA, USA).

#### 3.7.4. Label-Free Quantitative Proteomics Using Micro High-Performance Liquid Chromatography Coupled with Quadrupole Time-of-Flight Mass Spectrometry (Micro-HPLC-QTOF-MS)

##### Liquid Chromatography and Mass Spectrometry Setup

Label-free, bottom-up proteomic quantification was performed using a micro high-performance liquid chromatography system (Waters M-Class) coupled with a SCIEX™ TripleTOF^®^ 6600 quadrupole time-of-flight mass spectrometer (SCIEX, Framingham, MA, USA), operated in positive electrospray ionisation mode (ESI+). A total of 4 µg of tryptic peptide digest was injected onto a nanoEase M/Z HSS T3 column (1.8 µm, 300 µm × 150 mm; P/N 186009249) (Waters Australia Pty Ltd, Rydalmere, NSW 2116, Australia) with an in-line Zorbax 300SB-C18 guard column (5 µm, 5 × 0.3 mm; Agilent Technologies, Santa Clara, CA, USA). The column temperature was maintained at 40 °C. Mobile phase A consisted of 98% water and 2% acetonitrile, and mobile phase B consisted of acetonitrile with 0.1% formic acid. The system operated at a 5 µL/min flow rate, with loading and column washing steps conducted at 7 µL/min. The LC gradient was as follows: 2–10% B over 1.66 min at 7 µL/min, 10–25% B from 1.67 to 21.67 min at 5 µL/min, followed by a sharp increase to 95% B from 23.33 to 24.67 min, held for 2 min, and re-equilibrated at 2% B for 9 min at 7 µL/min.

##### Mass Spectrometry Acquisition Parameters

The mass spectrometer had a DuoSpray™ ion source and a 25 μm internal diameter electrode. Data were acquired using the Analyst 1.8.1 software suite and associated LC control drivers. Key ion source parameters were as follows: GS1 = 25, GS2 = 15, curtain gas = 20, ion spray voltage floating = 5500 V, and ion source temperature = 150 °C. The acquisition employed the SWATH™ data-independent acquisition (DIA) strategy, comprising an MS1 survey scan (*m*/*z* 350–1250, 50 ms accumulation) followed by 40 variable-width MS2 windows (*m*/*z* 400–1250), each with a 35 ms accumulation time, covering the full precursor *m*/*z* range. MS2 spectra were acquired in high-resolution mode across *m*/*z* 100–2000, with an overall cycle time of approximately 1.5 s.

##### Mass Calibration and Library Generation

PepCalMix calibrant; P/N 5045759; 10 fmol/µL (SCIEX, Framingham, MA, USA), was diluted 1:100 in 5% acetic acid and 2% acetonitrile and was injected every 12 samples to ensure mass accuracy. Six pooled quality control (QC) samples were used to construct a DIA-only spectral library using a gas-phase fractionation approach [154], covering the following *m*/*z* segments: 400–500, 500–600, 600–700, 700–800, 800–900, and 900–1000. The precursor isolation window was set to 5 *m*/*z*, with a collision energy spread of 5 eV, except for *m*/*z* 700–990 (8 eV) and 990–1000 (10 eV). Each DIA segment cycle time was 2.14 s, incorporating low- and high-energy scans with 40 ms MS2 accumulation.

##### Data Processing and Statistical Analysis

Data were processed using Spectronaut v19.5, which implemented the DirectDIA+ workflow with the Biognosys Standard (BGS) analysis framework. The canonical human reference proteome (UniProt, released 24 January 2024; 17,179 entries) was used as the reference database. Searches were conducted using Pulsar, with enzymatic specificity for trypsin/P and LysC/P, allowing up to 2 missed cleavages. Peptide lengths were restricted to 7–52 amino acids. Carbamidomethylation (C) was set as a fixed modification, while variable modifications included protein N-terminal acetylation, methionine oxidation, and methylation and demethylation. A maximum of five variable modifications per peptide was permitted. Peptide–spectrum matches (PSMs), peptides, and protein groups were filtered at a % false discovery rate (FDR) of 1%. Label-free quantification (LFQ) was performed automatically using MS2 area integration with default normalisation strategies. Protein inference employed the IDPicker algorithm.

Ingenuity Pathway Analysis (IPA, QIAGEN Digital Insights, Redwood City, CA, USA) was implemented for pathway enrichment analyses using differentially expressed proteins (DEPs at absolute Log_2_FC 0.58 and Q 0.05) to identify canonical pathways responsible for the anticancer mechanisms of monotreatment vs. control and the synergistic effects of the combinations vs. the monotreatments against the HKB-11 lymphoma cells.

##### Data Availability

The mass spectrometry proteomics data were deposited in the ProteomeXchange Consortium via the PRIDE [155] partner repository with the dataset identifier PXD063948.

### 3.8. Statistical Analysis

Data were collected and managed using MS Office Excel and GraphPad Prism for statistical analyses and visualisation. Data collection and analyses were carried out in triplicate, and the outcomes were presented as the mean ± standard deviation. Statistical significance between the mean values was determined at *p* ≤ 0.05 employing a two-way ANOVA. Tukey and Dunnett’s tests were utilised within GraphPad Prism software to perform nonlinear regression and multiple comparisons. Furthermore, GraphPad Prism software computed the IC_50_ value (representing the drug concentration required to achieve a 50% cell growth inhibition). The experimental groups in the proteomics study were compared statistically using unpaired *t*-tests, assuming equal variances. Candidate proteins were selected based on an absolute log_2_ fold change ≥ 0.58 and a Q-value ≤ 0.05. Enrichment analysis was subsequently conducted using Ingenuity Pathway Analysis (IPA), applying a significance threshold of adjusted Q ≤ 0.05 to identify functionally enriched pathways, focusing on those with an absolute z-score of ≥1.

## 4. Conclusions

This research provided robust evidence supporting the therapeutic potential of N and UB against lymphoma, with significant antiproliferative and pro-apoptotic effects individually and synergistically against the HKB-11 lymphoma cell line. Moreover, the monotherapies and their combination significantly outperformed in both the HKB-11 and Hs 313.T lymphoma cell lines, with selective cytotoxicity sparing HS-5 normal stromal cells at lower concentrations, suggesting a favourable therapeutic window. Furthermore, flow cytometry results revealed that a higher dose of UB (500 µM) was the most potent inducer of apoptosis. However, the combination of N and UB in a 4:6 ratio triggered a stronger apoptotic response than either monotherapy, N at 3200 and UB at 300 µM. Our findings demonstrated that while N can significantly elevate ROS at higher doses and potentially induce apoptosis in cancer cells, its combination with UB provides a balanced oxidative profile. The synergistic induction of apoptosis may result from complementary mechanisms, wherein N disrupts mitochondrial membranes and elevates ROS, while UB impairs mitochondrial respiration and modulates metabolic pathways. Together, these effects likely overwhelm lymphoma cells’ antioxidant defences and activate intrinsic apoptosis pathways. Additionally, proteomics analysis further indicated that N (3200 µM) treatment resulted in widespread downregulation of proteins essential for cell cycle progression and nucleotide metabolism, such as CDK4, PRC1, RFC1, PCNA, and TYMS. These changes reflected a potential blockade of cell cycle progression and DNA synthesis stages, promoting replication stress and apoptotic signalling. Additionally, the suppression of LARP1 and MAPK14 indicated probable impairment of mTOR signalling and cellular stress responses in the HKB-11 cells, further reducing proliferative potential. UB (300 µM) treatment induced a significant mitochondrial shift, upregulating components of the electron transport chain, including COX6C, MT-CO2, and NDUFA11, which suggests an enhanced oxidative phosphorylation capacity. Furthermore, this metabolic reprogramming likely supported energy-dependent survival under oxidative stress. However, it was counterbalanced by marked downregulation of mitotic checkpoint proteins, including MAD2L1, UBE2S, and UBE2E1, indicating potential mitotic arrest and increased genomic instability. Notably, the N and UB combination elicited a synergistic response, characterised by coordinated effects on metabolism, cell division, and immune evasion. Notable upregulation of NNMT indicated altered NAD^+^ metabolism and tumour metabolic stress in the HKB-11 cells. Additionally, the upregulation of A2M and SERPINF1 suggested enhanced immune-modulatory and anti-angiogenic effects, potentially limiting tumour invasion and vascularisation. Similarly, the suppression of BUB3, CCNB1, CDCA8, and CDK1 supported mitotic disruption and proliferative collapse in the HKB-11 lymphoma cells upon treatment with the N and UB combination. Although the proteomic analysis identified significant alterations such as CDK4 downregulation and COX6C upregulation, we acknowledge the value of orthogonal validation. Given the limitations of traditional antibody-based methods and the complexity of the targets, we believe such validation would be more biologically meaningful in advanced in vivo models where the tumour microenvironment and treatment context are preserved. In conclusion, these findings presented compelling evidence that postbiotic therapies, particularly the N and UB combination, could significantly advance research on lymphoma treatment. Although our data are promising, this study is limited by its exclusive reliance on in vitro lymphoma cell models. Further validation in physiologically relevant systems, including organoids and animal models (such as xenograft or syngeneic lymphoma models), is essential to evaluate the in vivo efficacy, pharmacokinetics, pharmacodynamics, and safety profile of the N and UB combination. Future studies should also explore optimal dosing, mechanisms of action in complex microenvironments, and the potential for translation into early-phase clinical trials. Overall, the combination therapy significantly enhanced cancer cell apoptosis, modulated oxidative stress, and induced substantial proteomic changes in key cancer-related pathways, including lipid metabolism, mitochondrial respiration, and cell cycle regulation, in the HKB-11 lymphoma cell line. 

## Figures and Tables

**Table 1:** Antiproliferative activity of seven gut microbial postbiotics against HKB-11 human lymphoma cells. Cell growth inhibition was measured after 72 h of treatment using the Alamar Blue assay. The postbiotics tested were Nisin (N), sodium butyrate, sodium propionate, magnesium acetate, inosine, Urolithin A (UA), and Urolithin B (UB). Data are presented as the mean percentage of growth inhibition ± standard deviation (*n* = 9). Within each concentration (row), values with different superscript letters (a, b, c, d, e) are significantly different from each other (*p* < 0.05, ANOVA with Tukey’s post hoc test). IC_50_ values represent the concentration required to inhibit 50% of cell growth. “ND” indicates no activity detected; “NA” indicates not analysed. The results show that Urolithin B was the most potent antiproliferative compound, followed by Urolithin A and Nisin.

Cell Growth Inhibition (%) of HKB-11 Lymphoma Cell Line								
Concentration (µM)	N	Sodium Butyrate	Sodium Propionate	Magnesium Acetate	Inosine	Concentration (µM)	UA	UB
16,000	NA	63.83 ± 8.99 ^a^	51.45 ± 12.41 ^b^	18.23 ± 5.66 ^c^	35.2 ± 10.76 ^d^	500	61.51 ± 13.44 ^a^	81.81 ± 9.51 ^b^
8000	100.26 ± 0.09 ^a^	57.83 ± 9.95 ^b^	38.07 ± 10.39 ^c^	9.97 ± 3.12 ^d^	30.91 ± 6.27 ^e^	250	39.31 ± 12.18 ^a^	74.24 ± 4.91 ^b^
4000	100.21 ± 0.18 ^a^	53.18 ± 10.22 ^b^	16.31 ± 7.30 ^c^	9.86 ± 4.18 ^d^	27.02 ± 8.27 ^e^	125	18.22 ± 10.15 ^a^	55.51 ± 7.58 ^b^
2000	65.22 ± 1.35 ^a^	45.36 ± 9.08 ^b^	11.82 ± 2.51 ^c^	8.57 ± 4.41 ^d^	19.32 ± 7.15 ^e^	62.5	12.03 ± 5.16 ^a^	39.63 ± 7.48 ^b^
1000	34.84 ± 3.52 ^a^	23.33 ± 6.66 ^b^	10.26 ± 3.23 ^c^	7.85 ± 4.89 ^d^	14.41 ± 5.2 ^e^	31.25	10.77 ± 6.16 ^a^	36.16 ± 11.92 ^b^
500	15.18 ± 6.98 ^a^	18.58 ± 3.59 ^b^	10.99 ± 2.83 ^c^	7.61 ± 5.66 ^d^	13.54 ± 4.57 ^e^	15.625	8.16 ± 5.42 ^a^	30.96 ± 15.63 ^b^
250	2.64 ± 3.47 ^a^	15.31 ± 3.23 ^b^	9.47 ± 2.61 ^c^	6.82 ± 6.07 ^d^	12.42 ± 6.04 ^e^	7.8125	6.84 ± 4.75 ^a^	23.05 ± 2.71 ^b^
125	ND	13.38 ± 3.51 ^a^	7.75 ± 4.79 ^b^	6.66 ± 4.01 ^c^	9.45 ± 4.76 ^d^	3.90625	5.72 ± 5.12 ^a^	17.99 ± 3.26 ^b^
62.5	ND	11.64 ± 3.91 ^a^	5.7 ± 4.21 ^b^	5.47 ± 3.16 ^c^	7.69 ± 5.33 ^d^	1.953125	5.64 ± 5.61 ^a^	12.22 ± 2.73 ^b^
IC_50_	1467 µM	2022 µM	14597.14 µM	NA	NA	IC_50_	384.41 µM	87.56 µM

All cell growth inhibition (%) values are means ± standard deviations. ^a,b,c,d,e^ Values in the same row not having the same superscript letter are significantly different (*p* ≤ 0.05) from each other at the same concentration. “NA” indicates not analysed. “ND” indicates no activity detected.

**Table 2:** Synergistic interactions between Nisin (N) and Urolithin B (UB) in HKB-11 lymphoma cells. Cells were treated with nine different fixed-ratio combinations of Nisin and Urolithin B for 72 h.

Combinations N/UB	IC_50_	IC_75_	IC_90_	IC_95_
1:9 (800:450 μM)	1.02	0.74	0.54	0.44
2:8 (1600:400 μM)	1.03	0.88	0.76	0.69
3:7 (2400:350 μM)	0.95	0.61	0.40	0.31
**4:6 (3200:300 μM)**	**0.77**	**0.33**	**0.15**	**0.09**
5:5 (4000:250 μM)	0.94	0.53	0.31	0.22
6:4 (4800:200 μM)	1.09	0.66	0.42	0.32
7:3 (5600:150 μM)	0.91	0.46	0.25	0.17
8:2 (6400:100 μM)	0.61	0.41	0.30	0.26
9:1 (7200:50 μM)	1.32	0.92	0.70	0.61

The combination index (CI) was calculated using the Chou–Talalay method to determine the nature of the interaction at different levels of effect (IC_50_, IC_75_, IC_90_, IC_95_). CI values < 1 indicate synergy, CI = 1 indicates an additive effect, and CI > 1 indicates antagonism. Bolded numbers highlight synergistic interactions (CI < 1). The 4:6 ratio of N/UB (3200 µM/300 µM) exhibited the strongest synergy, particularly at high effect levels (CI = 0.09 at IC_95_).

**Table 3:** Cell growth inhibition (%) against HKB-11 and Hs 313.T lymphoma cell lines and cell viability (%) of HS-5 normal stromal cell line at different concentrations of N/UB (4:6).

Concentration (μM) N/UB 4:6 (3200:300)	Cell Growth Inhibition (%)		Cell Viability (%)
	HKB-11	Hs 313.T	HS-5
3500	98.49 ± 2.43 ^a^	100.55 ± 0.04 ^a^	10.08 ± 4.01
1750	65.77 ± 9.03 ^a^	90.26 ± 1.17 ^b^	17.92 ± 2.52
875	46.80 ± 1.32 ^a^	86.33 ± 1.59 ^b^	23.46 ± 3.96
437.5	30.04 ± 7.51 ^a^	76.06 ± 4.43 ^b^	66.37 ± 11.53
218.75	25.74 ± 17.07 ^a^	29.84 ± 7.04 ^a^	89.08 ± 9.20
109.375	21.86 ± 16.07 ^a^	9.99 ± 8.81 ^a^	92.32 ± 7.72
IC_50_	1304 μM	335.4 μM	551.6 μM

Data are presented as mean ± standard deviation (SD). ^a,b^ Values in the same row not having the same superscript letter are significantly different (*p* ≤ 0.05) from each other at the same concentration.

## Data Availability

The original contributions presented in this study are included in the article and Supplementary Materials. Further inquiries can be directed to the corresponding author.

## Supplementary Materials

The following supporting information can be downloaded at https://www.mdpi.com/article/10.3390/ijms26146829/s1.
